# CTX-M-15-Producing *E. coli* Isolates from Food Products in Germany Are Mainly Associated with an IncF-Type Plasmid and Belong to Two Predominant Clonal *E. coli* Lineages

**DOI:** 10.3389/fmicb.2017.02318

**Published:** 2017-11-21

**Authors:** Alexandra Irrgang, Linda Falgenhauer, Jennie Fischer, Hiren Ghosh, Elisabet Guiral, Beatriz Guerra, Silvia Schmoger, Can Imirzalioglu, Trinad Chakraborty, Jens A. Hammerl, Annemarie Käsbohrer

**Affiliations:** ^1^Department Biological Safety, German Federal Institute for Risk Assessment, Berlin, Germany; ^2^Institute of Medical Microbiology, German Center for Infection Research, Partner Site Giessen-Marburg-Langen, Justus Liebig University, Giessen, Germany; ^3^Barcelona Institute for Global Health (ISGlobal), Hospital Clínic-Universitat de Barcelona, Barcelona, Spain; ^4^European Food Safety Authority, Parma, Italy; ^5^Institute of Veterinary Public Health, Department for Farm Animals and Veterinary Public Health, University of Veterinary Medicine, Vienna, Austria

**Keywords:** antimicrobial resistance, CTX-M-15, livestock, genome, plasmid, distribution, ESBL

## Abstract

Extended-spectrum beta-lactamases (ESBL) mediating resistance to 3rd generation cephalosporins are a major public health issue. As food may be a vehicle in the spread of ESLB-producing bacteria, a study on the occurrence of cephalosporin-resistantu *Escherichia coli* in food was initiated. A total of 404 ESBL-producing isolates were obtained from animal-derived food samples (e.g., poultry products, pork, beef and raw milk) between 2011 and 2013. As CTX-M-15 is the most abundant enzyme in ESBL-producing *E. coli* causing human infections, this study focusses on *E. coli* isolates from food samples harboring the *bla*_CTX-M-15_ gene. The *bla*_CTX-M-15_ gene was detected in 5.2% (*n* = 21) of all isolates. Molecular analyses revealed a phylogenetic group A ST167 clone that was repeatedly isolated from raw milk and beef samples over a period of 6 months. The analyses indicate that spread of CTX-M-15-producing *E. coli* in German food samples were associated with a multireplicon IncF (FIA FIB FII) plasmid and additional antimicrobial resistance genes such as *aac(6)-Ib-cr, bla*_OXA−1_, *catB3*, different *tet*-variants as well as a class 1 integron with an *aadA5/dfrA17* gene cassette. In addition, four phylogenetic group A ST410 isolates were detected. Three of them carried a chromosomal copy of the *bla*_CTX-M-15_ gene and a single isolate with the gene on a 90 kb IncF plasmid. The *bla*_CTX-M-15_ gene was always associated with the IS*Ecp1* element. In conclusion, CTX-M-15-producing *E. coli* were detected in German food samples. Among isolates of different matrices, two prominent clonal lineages, namely A-ST167 and A-ST410, were identified. These lineages may be important for the foodborne dissemination of CTX-M-15-producing *E. coli* in Germany. Interestingly, these clonal lineages were reported to be widely distributed and especially prevalent in isolates from humans and livestock. Transmission of CTX-M-15-harboring isolates from food-producing animals to food appears probable, as isolates obtained from livestock and food samples within the same time period exhibit comparable characteristics as compared to isolates detected from human. However, the routes and direction of transmission need further investigation.

## Introduction

Resistance to 3rd generation cephalosporins in bacterial pathogens is of great concern in human medicine, since treatment options become increasingly limited in infections caused by multidrug-resistant Enterobacteriaceae. The most common resistance mechanisms in 3rd generation cephalosporin-resistant Enterobacteriaceae is the production of beta-lactamases (ESBL, AmpC and carbapenemases). The emergence and dissemination of ESBL-producing Enterobacteriaceae is mainly driven by horizontal gene transfer, especially conjugation/mobilization, as the enzymes are usually encoded on plasmids (Bonnet, 2004; Carattoli, 2013). Epidemic plasmids, which are detected amongst farm and companion animals, food and humans, belong to the incompatibility groups (Inc.,) F, A/C, N, HI2, I1 and K (EFSA Panel on Biological Hazards, 2011). However, increasing reports of chromosomal localization of antibiotic resistance genes, indicates that spread of the cephalosporin resistance might also be mediated via clonal spread (Hirai et al., 2013; Price et al., 2013; Rodriguez et al., 2014). The ESBL/AmpC genes are sometimes flanked by mobile genetic elements (e.g., transposons, IS elements or class 1 integrons), which are also responsible for successful transmission, and in case of IS*Ecp1* and IS*CR1*, also involved in the expression of the genes (Poirel et al., 2008).

ESBL-producing isolates are frequently reported from samples of livestock origin. Spread and persistence has been demonstrated in different studies (Carattoli, 2009; Liebana et al., 2013). Transmission of ESBL/AmpC-producing *Escherichia coli* from animal to humans is assumed. Contaminated food as a transmission vehicle is often discussed, but direct evidence to support this hypothesis is rare (Leverstein-van Hall et al., 2011). Often, transmission is suggested by indirect evidence through the detection of similar clones, plasmids or sequence types in different populations (EFSA Panel on Biological Hazards, 2011). In Germany, infections with ESBL-producing *E. coli* in humans are most commonly associated with CTX-M-15 enzymes, followed by CTX-M-1, -14 and -27 (Ewers et al., 2012; Valenza et al., 2014; Falgenhauer et al., 2016a; Pietsch et al., 2017). In contrast, the most common ESBL-type in animals is CTX-M-1, whereas CTX-M-15 is underrepresented in samples from animal livestock in European countries (EFSA Panel on Biological Hazards, 2011; Day et al., 2016). Similar observations were also made in food. Studies from Germany on chicken meat revealed that the most detected ESBL enzymes belonged to the CTX-M-1 type or SHV (Kola et al., 2012; Campos et al., 2014). Neither in these studies nor in a comprehensive study on ESBL in food from the UK, CTX-M-15 enzymes could be detected (Randall et al., 2017). Nevertheless, *bla*_CTX-M-15_-encoding *E. coli* from animal sources in Europe have been described (Lopez-Cerero et al., 2011; Valentin et al., 2014). The risk of contaminated food for the consumers was clearly shown within the German EHEC-outbreak in 2011 caused by the consumption of fenugreek sprouts contaminated with CTX-M-15-producing *E. coli* O104:H4 clone (Beutin and Martin, 2012; Weiser et al., 2013).

One of the aims of the German national research consortium RESET (2011–2016) was to reveal possible transmission pathways for ESBL/AmpC-producing Enterobacteriaceae. Harmonized protocols were established for the isolation of phenotypically cephalosporin-resistant bacteria from livestock, environment, food, companion animals, and humans to generate a comparable set of data. A previous study on CTX-M-15-producing *E. coli* of livestock origin (Fischer et al., 2014) found a frequent occurrence of isolates belonging to the clonal complex 10 (CC10), as well as clonal spread of ST410 isolates. Supporting this, a phylogenetic analysis based on whole genome data of CTX-M-15-producing isolates obtained from German livestock, companion animals, humans and environment was carried out, revealing interspecies dissemination of ST410 clones (Falgenhauer et al., 2016a). In the present study, CTX-M-15-producing *E. coli* isolated from animal food samples of different matrices were taken in the same period (2011–2013) as the livestock samples and were comprehensively characterized.

## Materials and methods

### Bacterial isolates and cultivation

More than 2,500 food samples of different origins (poultry, cattle, swine, vegetables) and matrices (meat and meat preparations, raw milk, cheese, vegetables) were taken by official food inspectors and investigated by German state laboratories (Saxony, Lower Saxony, Hesse, Bavaria). Food samples from processing plants, retail and raw milk samples were collected at the farm level. From each sample, 25 g were investigated by a non-selective pre-enrichment step for 18–24 h at 37°C in lysogeny broth (LB) following selective cultivation of 10 μl aliquots on MacConkey agar supplemented with 1 mg/L cefotaxime (CTX, Sigma-Aldrich, Munich, Germany) for 18–24 h at 37°C. The identification of *E. coli* was confirmed by MALDI-TOF (Biotyper, Bruker). From each sample one *E. coli* isolate phenotypically resistant to CTX was sent to the German National Institute for Risk Assessment (BfR). Positive samples were obtained from all analyzed matrices, even though only raw milk cheese was burdened and only one vegetable sample was tested positive. The ESBL genotype of 437 isolates was verified by PCR and Sanger sequencing as previously described (Rodriguez et al., 2009). Isolates positive for *bla*_CTX-M-15_ were included in this study and further characterized. Phylogenetic groups were classified as previously described (Doumith et al., 2012). The antimicrobial resistance pattern was determined by microbroth dilution according to CLSI guidelines (CLSI M07-A9) at the National Reference Laboratory for Antimicrobial Resistance (NRL-AR, BfR). The used antimicrobial panel was in concordance to the decision 2013/652/EU of the European Union commission and was carried out with microtiter plates from TREK Diagnostic Systems (Thermo Fisher Scientific, Schwerte, Germany).

### Molecular typing and characterization

Molecular characterization was performed using pulsed-field gel-electrophoresis (PFGE) and multi-locus sequence typing (MLST). Phylogenetic relationship of the isolates was determined using XbaI-PFGE analysis according to the PulseNet protocol (https://www.cdc.gov/pulsenet/pathogens/protocols.html). MLST was performed using the Achtman scheme (*adk, fumC, gyrB, icd, mdh, purA, recA*; http://mlst.warwick.ac.uk/mlst/dbs/Ecoli).

The location of the *bla*_CTX-M-15_ gene was determined by S1-nuclease PFGE (1–25 s 17 h, 120°, 6 V/cm) with subsequent southern blot hybridization using a *bla*_CTX-M-15_ PCR-probe (Rodriguez et al., 2009). A chromosomal location of the *bla*_CTX-M-15_ gene was assumed for isolates in which no positive signal had been detected. Plasmids harboring the *bla*_CTX-M-15_ gene were isolated by alkaline lysis and transformed into *E. coli* DH10B™ competent cells (Invitrogen™, Thermo Fisher Scientific, Schwerte, Germany) (Birnboim and Doly, 1979; Rodriguez et al., 2009). Selection of transformed cells was carried out on LB agar supplemented with 1 mg/L CTX. Transformation of plasmids was confirmed by PCR. Incompatibility groups of the transferred plasmids were determined by PCR using the PBRT kit (Diatheva, Cartoceto PU, Italy). When transformation experiments were inconclusive, incompatibility group of the *bla*_CTX-M-15_ harboring plasmid was determined using PFGE/southern blot hybridization with probes specific for IncF and IncI1. The IS*Ecp*1 element was detected by using modified ALA3/ALA4 Primer (5′-TTTGCGCATACAGCGGCACAC-3′/5′-CTATCCG**T**ACAAGGGAG-3′) (Rodriguez et al., 2014).

### Next generation sequencing (NGS) and *in silico* analyses

Additionally, whole genome sequencing of the isolates was performed. Therefore, genomic DNA was isolated from overnight cultures using the PureLink^®;^ Genomic DNA Mini Kit (Thermo Fisher Scientific, Schwerte, Germany). A NexteraXT library was generated and sequenced on a MiSeq benchtop sequencer (Illumina, CA, USA) with 2 × 300 bp paired-end reads. Raw reads were assembled using SPAdes (v 3.5.0) (Bankevich et al., 2012). Whole-genome-based phylogenetic analysis was performed using HarvestSuite (ParSNP) (Treangen et al., 2014).

Resistance genes, virulence genes, serotype and pMLST were predicted using the web-based tools of the Center for Genomic Epidemiology (Zankari et al., 2012; Carattoli et al., 2014; Joensen et al., 2014, 2015).

### Accession numbers

Whole genome sequences of the isolates have been deposited in the European Nucleotide Archive (ENA). Accession numbers of isolates RL16, RL25, RL36, RL40, RL63, RL162, RL195, RL212, RL224, RL230, RL239, RL330, RL331, RL345, RL346, RL364, RL379, RL406-0, RL452,RL464, and RL465 are summarized in the Table S2.

## Results and discussion

### Persistence of the ST167 clone amongst german CTX-M-15 food isolates

From 437 *E. coli* isolates phenotypically resistant to 3rd generation cephalosporins obtained from animal derived food, 404 isolates were confirmed as ESBL/AmpC-producing bacteria. Of these, 21 (5.2%) isolates harbored the *bla*_CTX-M-15_ gene. This is in agreement with the observations, that while resistance to 3rd generation cephalosporins in Germany and other European countries is frequently mediated by CTX-M-15 enzymes in isolates from human origin, they are of low prevalence in bacteria from livestock (Pfeiffer et al., 2013; Brolund, 2014; Valentin et al., 2014). A comparable study from the UK even found no CTX-M-15-producing *E. coli* in food samples from animals and non-animal sources while there was an overall prevalence of ESBL-producing *E. coli* of 27.5% of the meat samples (Randall et al., 2017)

The main characteristics of the isolates is given in Table 1. There are distinct similarities regarding detected STs, pMLST of IncF plasmids, class 1 integrons or virulence between isolates obtained from food and animal origin. These results suggest a transmission from animal to food (Fischer et al., 2014).

**Table 1:** Overview of the characteristics of CTX-M-15-producing *E. coli* isolates obtained from food samples.

**Isolate no**.	**Source**	**Isolation date (federal state^a^)**	**Resistance phenotype/acquired resistance genes**	**Phylogenetic group**	**PFGE pattern^a^**	**MLST (Clonal complex)**	**CTX-M-15 -plasmid size**	**Inc., Group (pMLST)**	**Class 1 integron^b^**
RL16	raw milk	06/08/2012 (S)	AMP, CIP, FOT, NAL, SMX, TAZ, TET, TMP*/aac(6′)Ib-cr, aadA5, bla*_CTX-M-15_, *bla*_OXA-1_, *catB3-*like, *dfrA17, mph(A), sul1, tet(B)*	A	P1	ST167 (CC10)	160 kb	FII, FIA, FIB (F31:A4:B1)	1,664 kb/*dfrA17 aadA5*
RL25	turkey meat (steak)	24/08/2012 (H)	AMP, CHL, CIP, FOT, KAN, NAL, SMX, STR, TAZ, TET, TMP / *aadA5, aph(3′)-Ia, bla*_CTX-M-15_, *bla*_TEM−1B_, *catA1-*like, *dfrA17, mph(A)*,*strA, strB, sul1, sul2, tet(A)*	A		ST410 (CC23)	None^c^	–	1,664 kb/*dfrA17 aadA5*
RL36	beef (shoulder)	11/05/2012 (S)	AMP, CIP, FOT, SMX, STR, TET, TMP / *aac(3)-IId, bla*_CTX-M-15_, *bla*_TEM-1*B*_, *mph(A),strA, strB, sul2*,*tet(B)*	A		ST167 (CC10)	None^c^	–	
RL40	beef (chuck)	18/06/2012 (S)	AMP, CIP, FOT, NAL, SMX, TAZ, TET, TMP/*aac(6′)Ib-cr, aadA5, bla*_CTX-M-15_, *bla*_OXA-1_, *catB3-*like, *dfrA17, mph(A), sul1, tet(B)*	A	P1	ST167 (CC10)	160 kb	FII, FIA, FIB (F31:A4:B1)	1,664 kb/*dfrA17 aadA5*
RL63	raw milk	20/09/2012 (H)	AMP, CHL, CIP, FOT, GEN, KAN, NAL, SMX, STR, TAZ, TET, TMP/*aac(3)-IIa*,*aac(6′)Ib-cr, aadA2, aadA5, aph(3′)-Ia, bla*_CTX-M-15_, *bla*_OXA-1_, *bla*_TEM-1*B*_, *catB3, catA1, dfrA12, dfrA17, mph(A), sul1, sul2, strA*,*strB, tet(A), tet(B)*	A		ST744 (none)	165 kb	FII, FIA, FIB (F22:A1: B20)	two: 1,664 kb*dfrA17, aadA5;* ~1,900kb *dfrA12, aadA2*
RL162	ground beef	31/08/2012 (BAV)	AMP, CIP, FOT, NAL, SMX, STR, TAZ, TET / *bla*_CTX-M-15_, *bla*_TEM-1*B*_, *catA1, strA, strB, sul2, tet(A)*	A		ST448 (CC448)	80 kb	I1 (ST-31)	
RL195	beef	09/10/2012 (BAV)	AMP, FOT, KAN, STR, TAZ, TET/*aph(3′)-Ic-*like, *bla*_CTX-M-15_, *bla*_TEM-1*B*_, *strA*-like, *strB*,*tet(B)*-like	D		ST69 (CC69)	78 kb	I1 (ST-31)	
RL212	suckling pig (shoulder)	17/10/2012 (BAV)	AMP, CIP, FOT, NAL, SMX, STR, TAZ, TET, TMP/*aac(6′)Ib-cr, aadA5 ,bla*_CTX-M-15_, *bla*_OXA−1_, *catB3-*like, *dfrA17, mph(A), strA, strB, sul1, sul2, tet(A)*	A		ST410 (CC23)	90 kb	FII, FIA, FIB (F31:A4:B1)	1,664 kb/*dfrA17 aadA5*
RL224	pork	19/10/2012 (BAV)	AMP, FOT, SMX, STR, TAZ, TET, TMP / *bla*_CTX-M-15_, *bla*_TEM-1*B*_, *dfrA5, strA, strB, sul2, tet(A)*	A		ST101 (CC101)	48 kb	N (ST-3)	~700 bp/*dfrA5*
RL230	ground pork	15/11/2012 (H)	AMP, FOT, KAN, SMX, STR, TAZ, TET, TMP / *aph(3′)-Ic-*like, *bla*_CTX-M-15_, *bla*_TEM-1*B*_, *dfrA5, strA, strB, sul2-like, tet(B)*	B1		ST3321 (none)	90 kb	I1 (ST-31)	~700 bp/ d*frA5*
RL239	raw milk	14/11/2012 (BAV)	AMP, FOT, TAZ/*bla*_CTX-M-15_	A		ST2325 (none)	60 kb	I2 (NA)	
RL330	raw milk	17/12/2012 (S)	AMP, CIP, FOT, NAL, SMX, TAZ, TET, TMP /*aac(6′)Ib-cr ,aadA5, bla*_*CTX*−*M*−15_, *bla_OXA-1_, catB3-like, dfrA17, mph(A), sul1, tet(B)*	A	P1	ST167 (CC10)	160 kb	FII, FIA, FIB (F31:A4:B1)	1,664 kb/*dfrA17 aadA5*
RL331	raw milk	17/12/2012 (S)	AMP, CIP, FOT, KAN, NAL, SMX, TAZ, TET, TMP /*aac(6′)Ib-cr, aadA5, bla*_CTX-M-15_, *bla*_OXA-1_, *catB3*-like, *dfrA17, mph(A), sul1, tet(B)*	A	P1	ST167 (CC10)	160 kb	FII, FIA, FIB (F31:A4:B1)	1,664 kb/*dfrA17 aadA5*
RL345	turkey meat (schnitzel)	21/12/2012 (S)	AMP, CHL, CIP, FOT, KAN, NAL, SMX, STR, TAZ, TET, TMP/*aac(6′)Ib-cr*, a*adA1, aadA2-*like, *aadA5, bla*_*CTX*−*M*−15_, *bla*_*OXA*−1_, *bla*_TEM-1*C*_, *catB3*-like, *cmlA1*-like, *dfrA17, mph(A), strA-*like, *strB, sul1, sul2, sul3, tet(A)*	A	P2	ST167 (CC10)	150 kb	FII, FIA, FIB (F31:A4:B1)	1,664 kb/*dfrA17 aadA5*
RL346	turkey meat (schnitzel)	21/12/2012 (S)	AMP, CHL, CIP, FOT, KAN, NAL, SMX, STR, TAZ, TET, TMP/*aac(6′)Ib-cr, aadA1, aadA2, aadA5, bla*_*CTX*−*M*−15_, *bla*_OXA−1_, *bla*_TEM-1*C*_, *catB3, cmlA1, dfrA17, mph(A), strA, strB, sul1, sul2, sul3, tet(A)*	A	P2	ST167 (CC10)	190 kb	FII, FIA, FIB (F31:A4:B1)	1,664 kb/*dfrA17 aadA5*
RL364	raw milk	14/02/2013 (S)	AMP, CIP, FOT, NAL, SMX, TAZ, TET, TMP/*aac(6′)Ib-cr, aadA5, bla*_CTX-M-15_, *bla*_*OXA*−1_, *catB3-*like, *dfrA17, mph(A), sul1, tet(B)*	A	P1	ST167 (CC10)	160 kb	FII, FIA, FIB (F31:A4:B1)	1,664 kb/*dfrA17 aadA5*
RL379	pork (rib)	07/03/2013 (H)	AMP, CHL, CIP, FOT, NAL, SMX, STR, TAZ, TET/*bla*_CTX-M-15_, *catA1*-like, *strA, strB, sul2, tet(B)*	A		ST540 (none)	110 kb	FII, FIA, FIB (F1:A1:B49)	
RL406-0	chicken giblets	04/04/2013 (S)	AMP, CIP, FOT, NAL, SMX, STR, TAZ, TET, TMP*/aadA2, bla*_CTX-M-15_, *bla*_TEM-1*B*_, *dfrA12, mph(A), strA*-like, *strB*-like, *sul1, sul2, tet(A)*	A		ST410 (CC23)	None^c^	–	1,913 kb/*drfA12, aadA2*
RL452	ground beef	06/02/2013 (LS)	AMP, FOT, STR, TAZ, TET / *bla*_CTX-M-15_, *bla*_*TEM-*1*B*−_, *strA, strB, tet(B)*	B2		ST12 (CC12)	82 kb	I1 (ST-31)	
RL464	turkey meat (breast)	13/11/2013 (LS)	AMP, CHL, CIP, FOT, SMX, STR, TAZ/*aadA1, aadA2*-like, *bla*_CTX-M-15_, *bla*_TEM-135_, *cmlA1*-like, *qnrS1, sul3*	D		ST1140	105 kb	I1 (ST-36)	>4,000 kb/*sat psp aadA2, cmlA, aadA1*
RL465	turkey meat (breast)	18/11/2013 (LS)	AMP, CIP, COL, FOT, KAN, NAL, SMX, STR, TAZ, TET, TMP/*aadA1,aph(3′)-Ia, bla*_CTX-M-15_, *bla*_TEM-1*B*_, *dfrA1, mcr-1, strA*-like, *strB*-like, *sul2, sul3, tet(A)*-like	A		ST410 (CC23)	none^c^	-	~1,500 kb/*dfrA1, aadA1*

**Figure 1:**
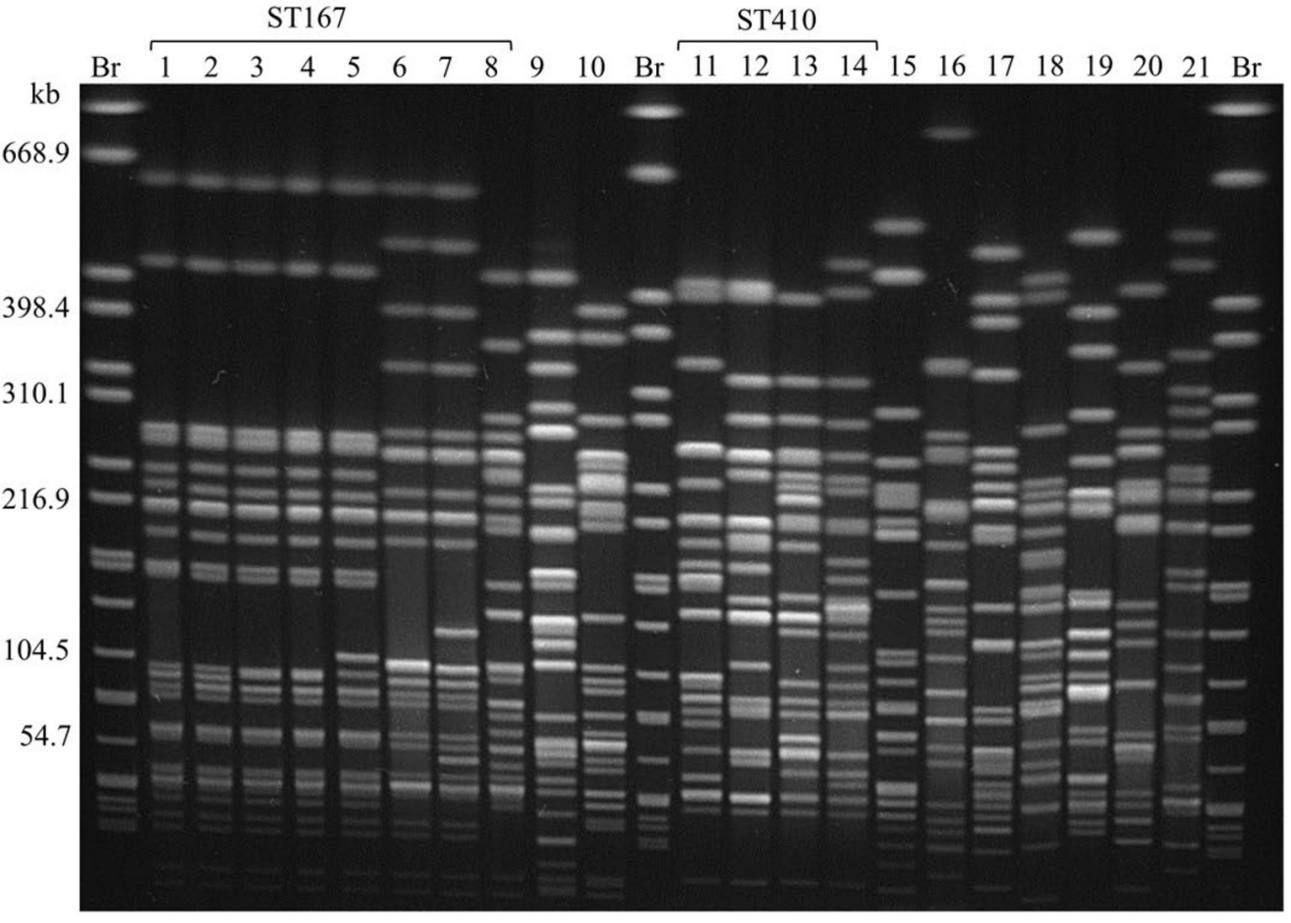
XbaI PFGE of *E. coli* isolates obtained from food harboring the *bla*_CTX−M−15_ gene. Br = *S*. Braenderup H9812, used as size standard. Lanes: **Table:** 

*Antimicrobials: AMP, ampicillin; CHL, chloramphenicol; CIP, ciprofloxacin; COL, colistin; FOT, cefotaxim; KAN, kanamycin; NAL, nalidixic acid; SMX, sulfamethoxazole; STR, streptomycin; TAZ, ceftazidim; TET, tetracycline; TMP, trimethoprim; Regions: S, Saxony; H, Hesse; LS, Lower Saxony; BAV, Bavaria*.

a *Please see Figure 1*.

b *PCR amplicons using CS5′/CS3′ Primers (Rodriguez et al., 2009) or identified by analyzing NGS data*.

c *Chromosomally located*.

There is a predominance of isolates belonging to clonal complex (CC) 10 (*n* = 8) and CC23 (*n* = 4) of the phylogenetic group A. Although the sequence types (ST) 38 and ST131 are typically observed in humans (Rodriguez et al., 2014),the frequent presence of ST167/ST617 (CC10) and ST410 (CC23) isolates from food samples in this study concurs with previous reports of isolates from animal samples within the same time period (2011–2013) and from human stool samples. In particular, those reported isolates harbored *bla*_CTX-M-15_ and were members of the same phylogenetic group (Fischer et al., 2014; Ben Sallem et al., 2015). This underlines a possible transmission from animals to humans via contaminated food.

**Figure 2:**
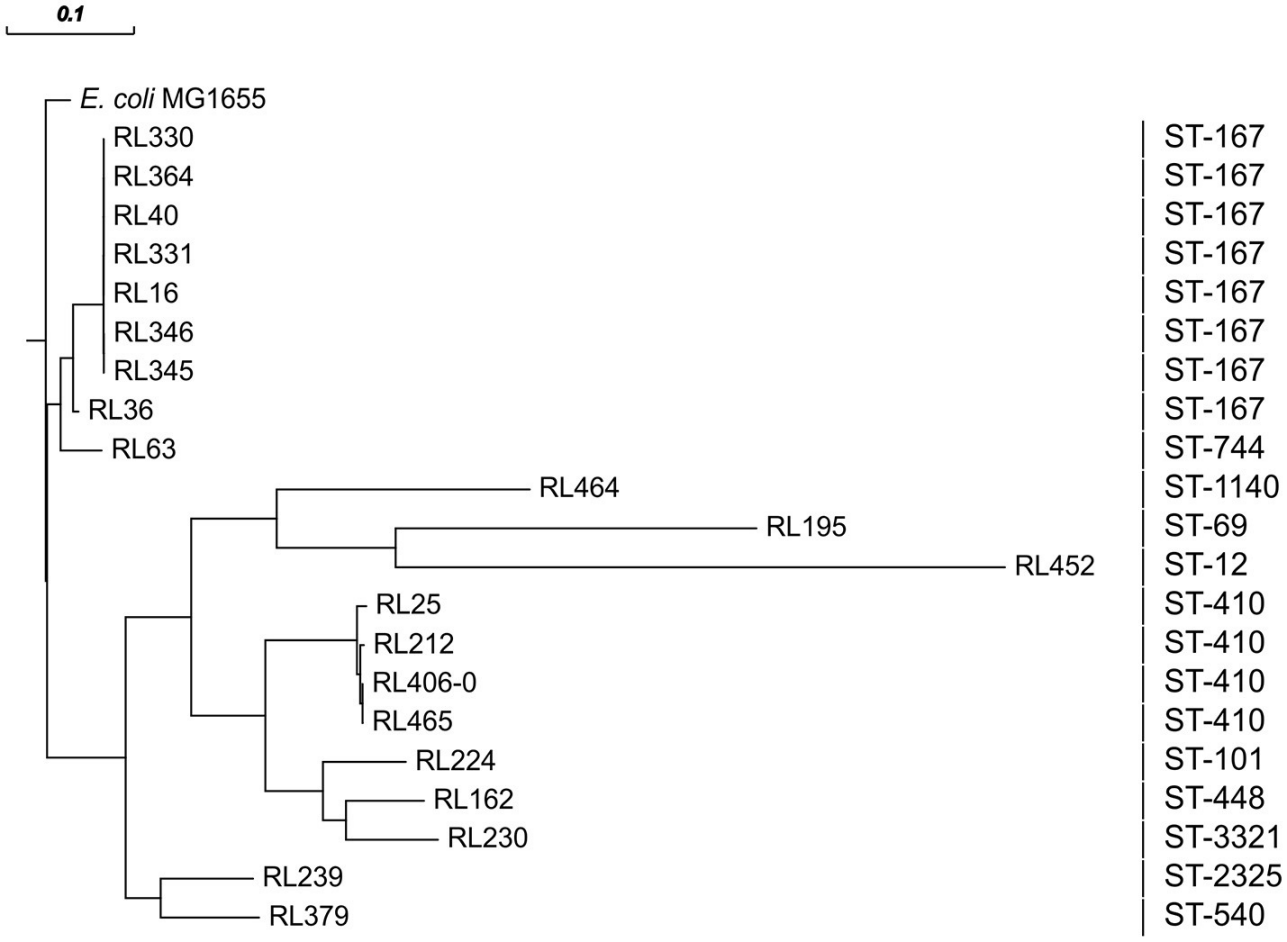
Phylogenetic analysis of all 21 sequenced CTX-M-15-producing isolates obtained from food performed by HarvestSuite (ParSNP), using *E. coli* MG1655 (NC_000913) as a reference. The scale defines the degree of the phylogenetic relationship of the isolates.

All CC10 isolates belong to ST167. Five of them showed an almost identical XbaI PFGE pattern (P1; Figure 1). These strains have been isolated over a period of 6 months from samples of raw milk (*n* = 4) and beef (*n* = 1) in Saxony (Eastern Germany). Isolates were obtained from four different samples taken at different time points from three different postal code locations. Milk samples were obtained from farms that were nearby, whereas the beef sample was taken about 200 km away. Therefore, a geographical spread of the clone might have occurred. In general, the phylogenetic group A clonal complex 10 (ST10/167/617) represents a successful clonal lineage, which can be found in humans, livestock, as well as in companion animals (Ewers et al., 2012). In this study two additional ST167 isolates obtained from turkey meat showed similar PFGE restriction patterns (P2; Figure 1) which are distinguishable from those of the raw milk isolates. Nevertheless, all eight ST167 isolates cluster when performing phylogenetic analysis based on whole genome sequences (Figure 2) and all isolates of this clade belong to the same serotype as shown by NGS data (Table S1).

1: RL16	2: RL40	3: RL330	4: RL331	5: RL364	6: RL345	7: RL346
8: RL36	9: RL162	10: RL63	11: RL25	12: RL212	13: RL406-0	14: RL465
15: RL195	16: RL452	17: RL239	18: RL224	19: RL230	20: RL2379	21: RL464

### Detection of a circulating ST410 clone and the impact of the chromosomal localization of the beta-lactamase gene

There are several isolates (*n* = 4) belonging to ST410 harboring mainly a chromosomal location of the *bla*_CTX-M-15_ gene. Only in one isolate (RL212) the *bla*_CTX-M-15_ was located on a 90 kb multireplicon IncF plasmid as described above. However, NGS data revealed a close phylogenetic relationship of all isolates (Figure 2). In the Supplementary Material, a comparison based on whole genome sequences with ST410 isolates from German livestock, companion animals, humans and environment is shown (Supplementary Figure 1). The food-related isolates can be found in three of the five clades (B, C, D), which otherwise comprise of isolates from farm or farm environment-related samples as well as samples of human origin. In concordance with the other strains of clade B, *bla*_CTX-M-15_ of RL25 integrated at a distinct location in the *rhs*E cassette, which is known as a hotspot for insertion sequences and recombination in *E. coli* (Saier, 2008). The *bla*_CTX-M-15_ of RL465 (Falgenhauer et al., 2016b) and RL406-0 also integrated at the same location known for the other members of clade C, at a defective lambdoid prophage region. The results of the current study further extend previous findings of interspecies circulation of ST410 clones to include food and point out the potential risk of contaminated food as transmission vehicles for consumers (Falgenhauer et al., 2016a).

Apart from the three ST410 isolates, a chromosomal localization is also likely for RL63 (ST176). The stable integration of the *bla*_CTX-M-15_ genes into the chromosome is reported for different MLST variants (Falgenhauer et al., 2016a). These findings demonstrate that the chromosomal integration of the *bla*_CTX-M-15_ gene occurred in several independent events and emphasize that a chromosomal location of this gene might be more common than anticipated (Rodriguez et al., 2014).

### *bla*_CTX-M-15_ is mainly located on plasmids of the incompatibility group IncF

Apart from strain RL36, where the *bla*_CTX-M-15_ seems to be located on the chromosome, the remaining seven ST167 isolates (PFGE pattern P1 and P2) harbor the *bla*_CTX-M-15_ gene on 150–190 kb multireplicon IncFIA/FIB/FII plasmids. These large IncF plasmids (>150 kb), as well as the ST410 IncF plasmid, additionally harbored an *aac(6)-1b-cr* gene (plasmid mediated quinolone resistance gene). The further correlation of *bla*_CTX-M-15_-encoding IncF plasmids with the detection of *bla*_OXA-1_, *catB3* and *tet* genes is also described by Lopez-Cerero et al. (2011). IncFII plasmids carrying *bla*_CTX-M-15_ are known to be highly transferable (Carattoli, 2009). Except for plasmids from RL63 and RL379, all multireplicon IncF plasmids of this study belong to the pMLST F31:A4:B1, indicating plasmid-related spread of *bla*_*CTX-M-*15_ carrying *E. coli* within different food production chains.

The *bla*_CTX-M-15_ - *aac(6)-1b-cr*- harboring IncF plasmids were also associated with an 1,664 kb large class 1 integron containing a *dfrA17/aadA5* gene cassette (Table 1) encoding for trimethoprim and aminoglycoside resistance. Similar class 1 integrons associated with *bla*_CTX-M-15_ of phylogenetic group A *E. coli* were detected in isolates of livestock and companion origin as well as in samples of healthy humans worldwide (Dureja et al., 2014; Fischer et al., 2014).

### Association of *bla*_CTX-M-15_ with mobile genetic elements

The *bla*_CTX-M-15_ gene was associated with an upstream located IS*Ecp1* element in all isolates, and has been frequently reported for *bla*_CTX-M-15_ positive isolates (Lartigue et al., 2004; Smet et al., 2010). The association with IS*Ecp1* was even detected for chromosomally encoded *bla*_CTX-M-15_ genes. This suggests that the resistance gene can be easily mobilized. Transposition of *bla*_CTX−M_ genes associated with the IS*Ecp1* element was demonstrated *in vitro* (Lartigue et al., 2004). The PCR for ISEcp1 was positive in all isolates except for one. For the isolate RL25 (ST410, chromosomal *bla*_CTX-M-15_) insertion event of an IS1-element into the *tnp*A gene (transposase encoding) was detected at identical position to those found in ST410 isolates of different origin in the same clade (Fischer et al., 2014; Falgenhauer et al., 2016a). IS*Ecp1* elements, which are truncated by different IS elements, are occasionally reported and their effects on mobilization and expression of *bla* genes as well as their role in plasmid evolution have been discussed (Smet et al., 2010; Alonso et al., 2017).

### Virulence associated genes amongst CTX-M-15 isolates

In addition, the occurrence of virulence genes in the isolates was examined (Table S1). Most of the food isolates contained relatively few virulence genes. These included bacteriocins, glutamate decarboxylase, capsule synthesizing enzymes and serum survival genes. These virulence associated genes were also recognized in CTX-M-15-producing isolates from animals (Fischer et al., 2014). One isolate (RL346) harbored *senB*, which encodes an enterotoxin, that is responsible for enterotoxic activity of enteroinvasive *E. coli* (EIEC) and *Shigella* spp. (Nataro et al., 1995). Another isolate (RL452) carried two toxin genes (*ncf1*–cytotoxic necrotizing factor, involved in urinary tract infection (Mills et al., 2000), and *vat*–vacuolating autotransporter toxin, known to mediate increased fitness of uropathogenic *E. coli* (UPEC) during systemic infections (Nichols et al., 2016). This isolate belonged to phylogenetic group B2. These findings support the general assumption of low pathogenic potential in isolates of phylogenetic group A and B1 (major phylogenetic groups detected in this study) as compared to the higher virulence properties in isolates of the phylogenetic group B2 and D.

## Conclusion

In conclusion, *bla*_CTX-M-15_ positive *E. coli* have been detected in ESBL-producing isolates obtained from food, albeit with a low prevalence. There are two major findings regarding the spread of these resistance genes in these isolates: (1) the *bla*_CTX-M-15_ can either be spread by successful IncF plasmids (pMLST: F31:A4:B1) or (2) it can be transmitted by clonal spread of ST410 isolates harboring a chromosomally encoded gene. This clone was also found in samples of animal and human origin within the same sampling period. Their virtual identity to animal-derived isolates indicates an animal origin of the isolates found in food samples, although cross-contamination cannot be ruled out. Independently, there is a risk for consumers related to exposure to ESBL genes by contaminated food, although a quantification of this issue is not possible. In future, the distribution of CTX-M-types should be closely monitored in a one-health approach, in particular by whole genome analysis of isolates, to detect actual trends and delineate dissemination pathways of the beta-lactamases.

## Author contributions

AK, TC, and BG designed the study. AI, JF, EG, and SS performed the experiments. AI, SS, LF, HG, and CI performed WGS-sequencing and bioinformatics. AI, LF, SS, and JAH analyzed the data. AI, JAH, and LF wrote the manuscript and prepared the tables and figures. All authors edited the manuscript.

### Conflict of interest statement

The authors declare that the research was conducted in the absence of any commercial or financial relationships that could be construed as a potential conflict of interest.
