# Supplementary material for: CTX-M-15-Producing E. coli Isolates from Food Products in Germany Are Mainly Associated with an IncF-Type Plasmid and Belong to Two Predominant Clonal E. coli Lineages
Source: Front Microbiol. 2017 Nov 21;8:2318. doi: 10.3389/fmicb.2017.02318 (PMC5702323; doi:10.3389/fmicb.2017.02318)
Supplement: Supplementary file 1 [file Table1.DOCX]

Supplementary Material

**CTX-M-15-producing *E. coli* isolates from food products in Germany are mainly associated with an IncF-type plasmid and belong to two predominant clonal *E. coli* lineages**

Alexandra Irrgang, Linda Falgenhauer, Jennie Fischer, Hiren Ghosh, Elisabet Guiral, Beatrice Guerra, Silvia Schmoger, Can Imirzalioglu, Trinad Chakraborty, Jens Andre Hammerl and Annemarie Käsbohrer

*** Correspondence:** Jens Andre Hammerl: jens-andre.hammerl@bfr.bund.de

# Supplementary Table:

Table S1: Virulence genes and serotype of CTX-M-15-producing isolates from food

| Isolate no. | Phylo-group | Virulence genes | Serotype |  |
| --- | --- | --- | --- | --- |
| RL16 | A | *capU, gad, iss* | O89:H9 |  |
| RL25 | A | *gad,lpfA* | O8:H21 |  |
| RL36 | A | *capU, gad, iss* | O89:H9 |  |
| RL40 | A | *capU,gad, iss* | O89:H9 |  |
| RL63 | A | *celb, gad* | O89:H9 |  |
| RL162 | A | *gad, espP, iha, lpfA, mchB, mchC, mchF* | O8:H8 |  |
| RL195 | D | *air, eilA, espP, gad, iss, lpfA* | 017/O44:H18 |  |
| RL212 | A | *gad,lpfA* | ONT:H9 |  |
| RL224 | A | *celb, gad, lpfA, iroN, mchF, iss, tsh* | O21:H21 |  |
| RL230 | B1 | *astA, gad, ireA, iroN, iss, lpfA, mchF* | ONT:H34 |  |
| RL239 | A | *gad,iss* | O66:H25 |  |
| RL330 | A | *capU ,gad, iss* | O89:H9 |  |
| RL331 | A | *capU ,gad, iss* | O89:H9 |  |
| RL345 | A | *capU ,gad, iss* | O89:H9 |  |
| RL346 | A | *capU, gad, iss, senB* | O89:H9 |  |
| RL364 | A | *capU, gad ,iss* | O89:H9 |  |
| RL379 | A | *gad* | O9:H30 |  |
| RL406-0 | A | *cma, gad, iroN, iss, lpfA* | O8:H9 |  |
| RL452 | B2 | *cnf1,gad,iroN,iss,mchB,mchC,mchF,mcmA,vat* | O4:H5 |  |
| RL464 | D | *gad* | O38:H39 |  |
| RL465 | A | *cma, gad,iroN, iss, lpfA* | O8:H9 |  |

Table S2: Accession numbers for isolates investigated in this study

| **Name** | **Sample** | **Sample secondary** | **Experiment** | **Run** |
| --- | --- | --- | --- | --- |
| RL16 | ERS1912963 | SAMEA104287985 | ERX2173577 | ERR2116719 |
| RL25 | ERS1912964 | SAMEA104287986 | ERX2173578 | ERR2116720 |
| RL36 | ERS1912965 | SAMEA104287987 | ERX2173579 | ERR2116721 |
| RL40 | ERS1912966 | SAMEA104287988 | ERX2173580 | ERR2116722 |
| RL63 | ERS1912967 | SAMEA104287989 | ERX2173581 | ERR2116723 |
| RL162 | ERS1912968 | SAMEA104287990 | ERX2173582 | ERR2116724 |
| RL195 | ERS1912969 | SAMEA104287991 | ERX2173583 | ERR2116725 |
| RL212 | ERS1912970 | SAMEA104287992 | ERX2173584 | ERR2116726 |
| RL224 | ERS1912971 | SAMEA104287993 | ERX2173585 | ERR2116727 |
| RL230 | ERS1912972 | SAMEA104287994 | ERX2173586 | ERR2116728 |
| RL239 | ERS1912973 | SAMEA104287995 | ERX2173587 | ERR2116729 |
| RL330 | ERS1912974 | SAMEA104287996 | ERX2173588 | ERR2116730 |
| RL331 | ERS1912975 | SAMEA104287997 | ERX2173589 | ERR2116731 |
| RL345 | ERS1912976 | SAMEA104287998 | ERX2173590 | ERR2116732 |
| RL346 | ERS1912977 | SAMEA104287999 | ERX2173591 | ERR2116733 |
| RL364 | ERS1912978 | SAMEA104288000 | ERX2173592 | ERR2116734 |
| RL379 | ERS1912979 | SAMEA104288001 | ERX2173593 | ERR2116735 |
| RL406-0 | ERS1912980 | SAMEA104288002 | ERX2173594 | ERR2116736 |
| RL452 | ERS1912981 | SAMEA104288003 | ERX2173595 | ERR2116737 |
| RL464 | ERS1912982 | SAMEA104288004 | ERX2173596 | ERR2116738 |
| RL465 | ERS1117675 | SAMEA3930541 | ERX1430695 | ERR1359224 |
