# Supplementary material for: CTX-M-15-Producing E. coli Isolates from Food Products in Germany Are Mainly Associated with an IncF-Type Plasmid and Belong to Two Predominant Clonal E. coli Lineages
Source: Front Microbiol. 2017 Nov 21;8:2318. doi: 10.3389/fmicb.2017.02318 (PMC5702323; doi:10.3389/fmicb.2017.02318)
Supplement: Supplementary file 2 [file DataSheet1.DOCX]

Supplementary Material

**CTX-M-15-producing *E. coli* isolates from food products in Germany are mainly associated with an IncF-type plasmid and belong to two predominant clonal *E. coli* lineages**

Alexandra Irrgang, Linda Falgenhauer, Jennie Fischer, Hiren Ghosh, Elisabet Guiral, Beatrice Guerra, Silvia Schmoger, Can Imirzalioglu, Trinad Chakraborty, Jens Andre Hammerl and Annemarie Käsbohrer

*** Correspondence:** Jens Andre Hammerl: jens-andre.hammerl@bfr.bund.de

# Supplementary Figure

**Supplementary Figure 1.** Phylogenetic analysis of ST410 CTX-M-15-producing isolates obtained from food (marked in blue) in comparison to isolates from animal, human and environment (previously published by Flagenhauer et al. 2016a) performed by HarvestSuite (ParSNP), using *E. coli* 789 (GenBank accession no. CP010315.1) as a reference.

Reference

Falgenhauer, Linda; Imirzalioglu, Can; Ghosh, Hiren; Gwozdzinski, Konrad; Schmiedel, Judith; Gentil, Katrin et al. (2016a): Circulation of clonal populations of fluoroquinolone-resistant CTX-M-15-producing *Escherichia coli* ST410 in humans and animals in Germany. In: *Int J Antimicrob Agents* 47 (6), S. 457–465. DOI: 10.1016/j.ijantimicag.2016.03.019.
